# Effect of intravenous oxycodone on the physiologic responses to extubation following general anesthesia

**DOI:** 10.1186/s12871-021-01350-5

**Published:** 2021-05-12

**Authors:** Menglu Jiang, Jiawei Ji, Xin Li, Zhenqing Liu

**Affiliations:** Department of Anesthesiology, Wuxi 9th Affiliated Hospital of Soochow University, No. 999 Liangxi Rd, 214062 Wuxi, China

**Keywords:** Intravenous oxycodone, Blood pressure, General anesthesia

## Abstract

**Background:**

Endotracheal intubation and extubation may cause undesirable hemodynamic changes. Intravenous oxycodone has recently been introduced and used for relieving hemodynamic alterations in response to intubation, but there is insufficient information regarding its application in stabilizing hemodynamics during extubation in the patients emerging from general anesthesia.

**Methods:**

One hundred patients, who had undergone assorted laparoscopic surgeries under general anesthesia, were randomly assigned to Control group (saline injection, 50 cases) and Study group (intravenous injection of 0.08 mg/kg oxycodone immediately after completion of the surgical procedure, 50 cases). Blood pressure, heart rate, blood oxygen saturation (SpO_2_) as well as blood concentrations of epinephrine, norepinephrine, and cortisol were recorded or measured immediately before extubation (T_0_), during extubation (T_1_), as well as one minute (T_2_), 5 min (T_3_), and 10 min after extubation (T_4_). In addition, coughing and restlessness, time of eye-opening, and duration from completing surgery to extubation as well as Ramsay Sedation Scale were analyzed.

**Results:**

Blood pressure and heart rate as well as blood concentrations of epinephrine, norepinephrine, and cortisol were significantly higher in the Control group compared with the Study group at the time of extubation as well as 1, 5, and 10 min after extubation (*P* < 0.05). When the patients emerged from general anesthesia, 70 % of the Control group had cough, which was significantly higher than that of Study group (40 %, *P* < 0.05). Significantly higher number of patients manifested restlessness in the Control group before (40 %) and after extubation (20 %) compared with that in the Study group (20 and 2 %, respectively, *P* < 0.05). In addition, patients of Control group had lower Ramsay score at extubation (1.7 ± 0.7) as well as 30 min after extubation (2.4 ± 0.9) compared to that of the patients of Study group (2.2 ± 0.9, and 3.0 ± 0.8, respectively, *P* = 0.003 and 0.001).

**Conclusions:**

Intravenous oxycodone attenuated alterations of hemodynamics and blood hormones associated with extubation during emergence from general anesthesia.

**Trial registration:**

Chinese Clinical Trial Registry: ChiCTR2000040370 (registration date: 11-28-2020) “‘retrospectively registered”.

## Background

Intubation and extubation may cause adverse stimulation that results in undesirable hemodynamic alterations such as increased blood pressure, heart rate, and respiratory rate [1, 2]. Unstable hemodynamics can lead to perioperative complications such as cerebral hemorrhage, cardiac arrhythmia or failure in patients with pre-conditions of cardiac or cerebral disease [3]. Therefore, pharmaceutical intervention has been used and studied to attenuate hemodynamic alterations during and after intubation and extubation [4]. In this regard, opioids including oxycodone have been used for suppressing the hemodynamic response to laryngoscopy and tracheal intubation [5–7].

Oxycodone (14-hydroxy-7,8-dihydrocodeinone) is a semisynthetic opioid and exerts its biologic effect through the µ, κ and δ opioid receptors [8, 9]. Because oxycodone exerts its effect within 5 min after intravenous injection and lasts at least for 4 h [10, 11], intravenous oxycodone has been used and studied for relieving acute postoperative pain [5], as well as for attenuating hemodynamic alterations in response to endotracheal intubation [5, 6, 12]. The effect of oxycodone on hemodynamics during extubation in the emergence from general anesthesia, however, has not been studied. The current study was designed to evaluate effect of oxycodone on the physiologic changes associated with extubation during emergence from general anesthesia.

## Methods

### Patients

Patients, who had undergone laparoscopic surgeries under general anesthesia from March 2018 through December 2019 in our hospital, were prospectively enrolled into this study. The protocol of this study was approved by the Ethic Committee of The Ninth People’s Hospital of Wuxi City (No. KT2020026) and a signed consent was obtained from each participant or legal guardian.

### Inclusion criteria

Patients with Class I or II of the American Society of Anesthesia (ASA) Physical Status and Classification System, and received laparoscopic surgeries including cholecystectomy, hernia repair, and removal of the uterine and uterine appendages under general anesthesia.

### Exclusion criteria

Patients who had contraindication for general anesthesia; severe heart, liver or kidney diseases; mental disorder or history of over-dose using opioid analgesics.

### General anesthesia

Propofol (2 mg/kg), fentanyl (3 µg/kg), and cis-atracurium (0.2 mg/kg) were used for general anesthesia induction. After the induction, patients received oxygenation and denitrogenation through face mask for 3 min (100 % FiO_2_ and 6 L/min) followed by endotracheal intubation. For maintenance of general anesthesia, the following medicines were given by infusion pump: propofol 4–12 mg/kg/h, remifentanil 0.0-0.2 µg/kg/min, continuous inhalation of 1.5–2.5 % sevoflurane, and intermittent cis-atracurium. Propofol, remifentanil, and sevoflurane were discontinued 5–10 min prior to skin closure.

### Grouping and treatment

Patients were randomly and double-blindly assigned into the Control group or Study group by a Random Number Table. Control group was injected with normal saline, and Study group was intravenously injected with oxycodone hydrochloride (0.08 mg/kg) immediately after completion of the surgery.

### Parameters for outcome evaluation

Blood pressure, heart rate, and blood oxygen saturation (SpO_2_) were recorded or measured immediately before extubation (T_0_), at extubation (T_1_), one minute after extubation (T_2_), 5 min after extubation (T_3_), and 10 min after extubation (T_4_). Blood samples were collected at the aforementioned time points from the arm that was not on intravenous infusion. Concentrations of blood hormones including epinephrine, norepinephrine, and cortisol were measured by ELISA using commercially available kit (IBL, Germany) and ELISA plate reader (BioTek, Winooski, VT, USA). In addition, coughing and restlessness, duration from completing surgery to eye-opening, and duration from completing surgery to extubation were recorded blindly by two experienced nurses. Restlessness was defined as the patient was unable to follow instruction and cooperate within 30 min following extubation. Coughing was scored as following: 0 = no cough; 1 = mild cough; 2 = multiple coughing but lasted shorter than 5 s; 3 = multiple coughing and lasted longer than 5 s. Ramsay Sedation Scale was assessed at extubation (T_1_) as well as 30 min after extubation and scored as following: 1 = restless; 2 = co-operative and oriented; 3 = could response to commands; 4 = calm and easy to be awakened; 5 = hard to be awakened; 6 =. Sleeping and could not be awakened.

### Statistical analysis

Discrete variables were expressed by frequency (%) and analyzed by Chi-square test or Fisher’s exact test. Shapiro-Wilk test was used to test normality of the continuous variables followed by Student’s *t* test for normally distributed data, and Wilcoxon rank sum test was for comparison of the data that were not normally distributed. Analysis of variance (ANOVA) with replicate measures were used for comparison between groups at various time points. SPSS 19.0 software was used to perform all analysis and *P* value < 0.05 was considered as significant.

## Results

Demographic information indicated that there were no significant differences between the Control group and Study group in terms of gender ratio, age, height, weight, and amount of the medicines used for general anesthesia (Table 1).

**Table 1:** Demographic information of the patients

	Study group(*N* = 50)	Control group (*N* = 50)	c^2^/t	*P* value
Gender (M/F)	26/24	25/25	0.040	0.841
Age (y)	38.5 ± 6.5	39.2 ± 6.0	0.730	0.435
Height (cm)	169.1 ± 4.5	168.9 ± 4.8	0.212	0.753
Weight (kg)	63.2 ± 4.8	61.8 ± 5.6	0.232	0.737
Operation (min)	89.0 ± 13.6	95.0 ± 11.7	1.535	0.167
Remifentanil (µg/kg)	7.7 ± 0.5	7.8 ± 0.3	1.213	0.228
Propofol (mg/kg)	4.8 ± 0.4	4.7 ± 0.4	1.250	0.214

While blood pressure (both systolic and diastolic blood pressure) and heart rate were not significantly different in the two groups before extubation, they were significantly higher in the Control group compared to the patients treated with oxycodone hydrochloride (Study group) at the time of extubation (T_1_) and all subsequent measurement time points (*P* < 0.05, Table 2).

**Table 2:** Comparison of blood pressure, heart rate and oxygen saturation

Group	N	Time	SBP(mmHg)	DBP(mmHg)	HR(beats/m)	S_P_O_2_(%)
Control	50	T_0_	139.1 ± 31.3	76.1 ± 13.5	80.5 ± 13.8	96.5 ± 2.9
		T_1_	136.1 ± 18.2^*^	86.1 ± 11.3^*^	81.7 ± 8.1^*^	99.0 ± 1.0
		T_2_	167.6 ± 18.8^*^	91.7 ± 10.2^*^	93.8 ± 9.9^*^	97.9 ± 1.9
		T_3_	164.8 ± 17.7^*^	87.6 ± 10.4^*^	91.5 ± 8.1^*^	97.2 ± 2.0
		T_4_	148.7 ± 16.2^*^	89.9 ± 12.8^*^	87.5 ± 10.6^*^	98.1 ± 1.7
Study	50	T_0_	134.4 ± 26.6	79.9 ± 11.5	78.9 ± 12.2	97.1 ± 1.9
		T_1_	131.3 ± 18.8	82.3 ± 7.9	77.9 ± 11.3	98.3 ± 1.2
		T_2_	128.5 ± 22.1	81.4 ± 10.3	82.2 ± 10.6	98.4 ± 1.3
		T_3_	137.3 ± 16.1	78.6 ± 9.1	77.9 ± 10.2	98.6 ± 1.5
		T_4_	131.1 ± 17.2	80.8 ± 9.4	79.1 ± 10.4	98.5 ± 1.4

T_0_: immediately before extubation; T_1_:during extubation; T_2_: one minute after extubation: T_3_: 5 min after extubation; T_4_: 10 min after extubation

*SBP* systolic blood pressure, *DBP* diastolic blood pressure, *HR* heart rate, *SpO*_2_ blood oxygen saturation

*P < 0.05 compared to study group at corresponding time point

Similarly, there was no significant difference in the blood levels of epinephrine, norepinephrine, and cortisol between the two groups before extubation. However, they were significantly lower in the patients treated with oxycodone hydrochloride at extubation and afterwards (*P* < 0.05, Table 3).

**Table 3:** Comparison of hormone levels in response to the stimulation

Group	N	Time	EP (pmol/L)	NE (pmol/L)	CORT (pmol/L)
Control	50	T_0_	703.9 ± 101.6	1124.3 ± 132.6	325.0 ± 89.8
		T_1_	852.4 ± 99.7^*^	1333.1 ± 142.2^*^	459.0 ± 92.2^*^
		T_2_	842.4 ± 98.8^*^	1292.9 ± 169.4^*^	449.7 ± 78.8^*^
		T_3_	838.5 ± 96.6^*^	1289.0 ± 130.3^*^	440.9 ± 76.3^*^
		T_4_	828.8 ± 95.3^*^	1286.5 ± 129.3^*^	428.5 ± 80.3^*^
Study	50	T_0_	701.2 ± 99.9	1132.2 ± 142.4	332.2 ± 99.6
		T_1_	719.9 ± 100.7	1146.6 ± 138.8	339.9 ± 100.3
		T_2_	708.6 ± 92.5	1135.5 ± 122.4	342.8 ± 92.7
		T_3_	704.3 ± 90.3	1132.2 ± 121.3	332.5 ± 86.9
		T_4_	704.3 ± 92.5	1128.3 ± 120.7	326.7 ± 78.3

T_0_: immediately before extubation; T_1_: during extubation; T_2_: one minute after ectubation: T_3_: 5 min after extubation; T_4_: 10 min after extubation

*EP* epinephrine, *NE* norepinephrine, *CORT* cortisol

*P < 0.05 compared to study group at corresponding time point

Next, we compared the coughing and restlessness in the two groups. As shown in the Table 4, during the emergence from general anesthesia, majority (70 %) of the patients in the Control group had cough while 40 % patients of the Study group had cough (*P* < 0.05); at extubation, 20 % patients in the Control group had cough and it was significantly higher than that of the Study group (10 %, *P* < 0.05). Similarly, at extubation, majority (80 %) of the patients in the Control group had “coughing score 3”, which was significantly higher than that of the patients in the Study group (20 %, *P* < 0.05). In addition, significantly higher number of the patients manifested restlessness in the Control group before (40 %) and after extubation (20 %) compared to that in the Study group (20 and 2 % before and after, respectively, *P* < 0.05). However, there was no significant difference in the emergence from general anesthesia for the patients in either group in terms of eye-opening time as well as the duration between emergence from general anesthesia and extubation (*P* > 0.05).

**Table 4:** Comparison of coughing and restlessness between the two groups

	Control (*N* = 50)	Study (*N* = 50)	c^2^/t	*P* Value
Awakening time (min)				
Eye-opening time	10.7 ± 5.4	12.6 ± 4.3	1.413	0.132
Extubation time	14.9 ± 6.7	17.7 ± 5.8	2.17	0.089
Coughing (%)				
At eye-opening	35 (70)*	20 (40)	9.091	0.003
Extubation	10 (20)	5 (10)	1.961	0.161
Coughing score				
2 at extubation (%)	10 (20)*	40 (80)	36.00	< 0.001
3 at extubation (%)	40 (80)*	10 (20)	36.00	< 0.001
Restlessness				
Before extubation	20 (40)*	10 (20)	4.762	0.029
After extubation	10 (20)*	1 (2)	-	0.008

Eye-opening time: the duration from completion of surgery to patient open his/her eye; extubation time: the duration from completion of surgery to extubation

*P < 0.05 compared to study group at corresponding time point

Comparison of Ramsay score between the two groups revealed that patients in the Control group had lower Ramsay score at extubation (1.7 ± 0.7) as well as 30 min after extubation (2.4 ± 0.9) compared to that of the patients in the Study group (2.2 ± 0.9, and 3.0 ± 0.8, respectively, *P* = 0.003 at extubation and *P* = 0.001 30 min after extubation).

## Discussion

In the current study, we demonstrated that oxycodone hydrochloride could significantly attenuate blood pressure (both systolic and diastolic) and heart rate at the time of extubation as well as at 1, 5 and 10 min after extubation in the patients who emerged from general anesthesia. Furthermore, oxycodone hydrochloride could decrease blood levels of epinephrine, norepinephrine, and cortisol at extubation as well as at 1, 5, and 10 min after extubation, respectively. Oxycodone could also significantly reduce incidence of coughing and restlessness before and after extubation. However, Ramsay score was higher in the patients treated with oxycodone. These findings suggested that oxycodone could stabilize hemodynamics presumably through reducing epinephrine, norepinephrine and cortisol, but have sedative effect on the patients.

Stable hemodynamic status (blood pressure and heart rate changes were less than 20 % of the baseline) is a basic requirement for general anesthesia not only during the operation, but also after the operation. However, blood pressure and heart rate were sensitive to stimulations such as intubation and extubation. It has been reported that oxycodone could efficiently prevent tracheal intubation-related hemodynamic instability [13]. In the current study, several patients had hemodynamic changes (blood pressure and/or heart rate) that exceeded 20 % of the baseline after 1 or 5 min extubation. However, incidence of the 20 % or more changes in hemodynamics from baseline was significantly higher in the Control group compared to that of Study group (data not shown), suggesting that oxycodone administration through intravenous injection immediately after completion of a surgical procedure could significantly stabilize blood pressure and heart rate at extubation as well as at 1, 5, and 10 min after extubation. In addition, it has been reported that administration of oxycodone could result in significantly higher incidence of oxygen desaturation compared with that of fentanyl in patients without pre-oxygenation [5]. In the current study, however, there was no significant alteration in oxygen saturation in the patients treated with either oxycodone or saline, suggesting oxycodone injection (5 mg) do not affect oxygen saturation in the patients emerging from general anesthesia.

It has been reported that as high as 0.269 mg/kg oxycodone might be used in order to completely prevent endotracheal intubation-related hemodynamic alteration in the patients [12]. However, adverse effects including hypotension, bradycardia, and respiratory depression could occur at this higher dose of oxycodone [12]. Thus, appropriate concentration of oxycodone and speed of intravenous injection must be carefully selected. In this regard, it has also been reported that the predictive intravenous oxycodone ED95 was 0.091 mg/kg in male patients [12]. In the current study, we used 0.08 mg/kg oxycodone in the Study group, which was very close to the aforementioned concentration of oxycodone ED95. Under this concentration of oxycodone, patients in the current study not only had less incidence of coughing, but also less occurrence of restlessness before, during, and after extubation. In addition, side effects of oxycodone such as nausea and vomiting, which are common adverse events with oxycodone [14], were not analyzed in this study.

Similar to other opioid, oxycodone might also cause sedation. It has been reported that oxycodone (0.2 mg/kg) caused the patients to wake up later than those in the group of patients treated with fentanyl (2 µg/kg) by approximately 85 s in average in a study by Lee et al. [5]. However, none of these patients experienced any problems in terms of delayed awakening during 24 h postoperative observation [5]. In the current study, it was found that oxycodone did not prolong the awakening time for the patients as assessed by either eye-opening time or duration between emergence from general anesthesia and extubation. However, Ramsay scores at and after extubation were significantly lower in the Control group compared with that of Study group. These findings further suggested that oxycodone concentration used in the current study was safe and appropriate, but may have significant sedative effect.

It is known that oxycodone has lower intrinsic activity of binding µ-type receptor, but a stronger analgesic effect than morphine [15, 16]. This is because oxycodone can stimulate the peripheral and central opioid receptors of the µ and κ types, and κ receptors play a crucial role in mediating the analgesic activity of oxycodone [15]. In addition, oxycodone has a better effect of inhibiting visceral pain [17], which may contribute to its effect of stabilizing hemodynamics. In the current study, we demonstrated that oxycodone significantly suppressed blood levels of epinephrine, norepinephrine and cortisol at extubation as well as at 1, 5, and 10 min after extubation, suggesting oxycodone might stabilize heart rate and blood pressure through preventing aforementioned hormones from increasing in response to uncomfortable stimulation such as endotracheal intubation and extubation.

There were several limitations in the current study. First, this study explored oxycodone as the sole opioid in stabilizing hemodynamic status during and after extubation in the patients emerging from general anesthesia. A comparison of oxycodone with other opioids that are normally used for the purpose will be further meaningful information for clinical application of oxycodone for stabilizing hemodynamics during extubation. Second, although we observed adverse effects of oxycodone, the time was short (up to 10 min after extubation), which may not be sufficient to observe all adverse events associated with oxycodone such as nausea and vomiting, because intravenous oxycodone exerts its effect in 3–5 min and lasts for 4 h [11, 18]. Third, while the Ramsay scores at extubation and 30 min after extubation were statistically different between the two groups, the differences were minimal and may not be clinically significant. Finally, we did not evaluate intraoperative hemodynamics, which may have impact on postoperative requirement for opioids. Therefore, results of this study require further cohort studies and discussion in the future to further evaluate the feasibility of oxycodone in stabilizing hemodynamics in response to extubation.

Taken together, the current study demonstrated that intravenous administration of oxycodone immediately after completion of a surgery under general anesthesia had significant effect in stabilizing hemodynamics during and after extubation in the patients emerging from general anesthesia. Oxycodone could increase patients’ tolerance to uncomfortable stimulation of endotracheal extubation.

## Data Availability

The datasets used or/and analyzed during the current study are available from the corresponding author on reasonable request.

## Abbreviation

SpO_2_: Oxygen saturation
